# Blunt trauma to the parotid gland in child

**DOI:** 10.4103/0971-9261.43034

**Published:** 2008

**Authors:** Amit Agrawal, K. B. Golhar, Sankalp Dwivedi, Nitish Baisakhiya, Pankaj Banode, Abhishek Sachchar

**Affiliations:** Department of Surgery, Datta Meghe Institute of Medical Sciences, Sawangi (Meghe), Wardha, Maharashtra, India; 1 Department of ENT, Datta Meghe Institute of Medical Sciences, Sawangi (Meghe), Wardha, Maharashtra, India; 2 Department of Radiology, Datta Meghe Institute of Medical Sciences, Sawangi (Meghe), Wardha, Maharashtra, India

**Keywords:** Parotid, salivary gland, trauma

## Abstract

Blunt trauma to the parotid gland is extremely rare and requires considerable force. We present a unique case in which a child sustained parotid injury without any associated injury to the facial skeleton, parotid gland and ductal structures and managed successfully. A literature search revealed that this type of injury has not been reported previously.

## INTRODUCTION

Because of presence of a thick capsule, and its anatomical location (situated behind the strong mandibular skeleton) parotid gland is rarely injured by blunt trauma.[1] The force required is considerable, and there is usually an associated skeletal injury, to the mandible or temperomandibular joint and in these instances, the trauma to the other, more vital structures becomes more important.[1–3] We present a unique case of blunt traumatic injury to the parotid gland in a child without any associated injury to the facial skeleton. A literature search revealed that in children this type of injury has not been reported previously.

## CASE HISTORY

**Figure 1:**
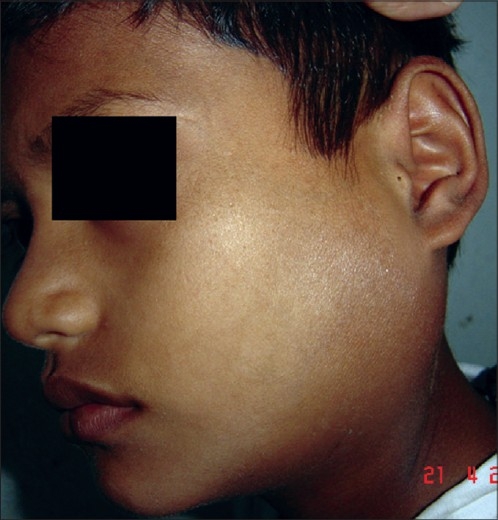
Clinical photograph showing swelling in the left parotid region with intact but shiny skin

**Figure 2:**
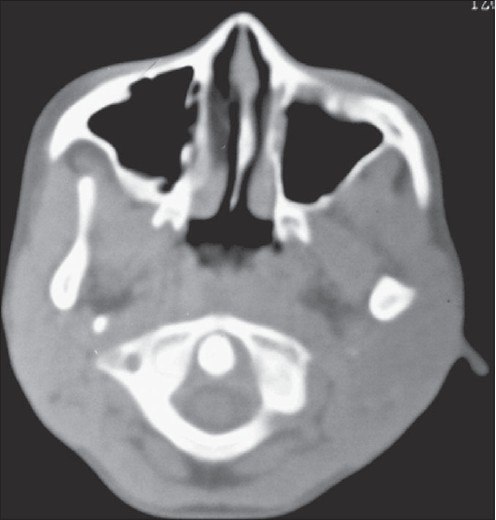
Computed tomography (bone window) showing swelling of the left parotid gland and no evidence of bony injury

**Figure 3:**
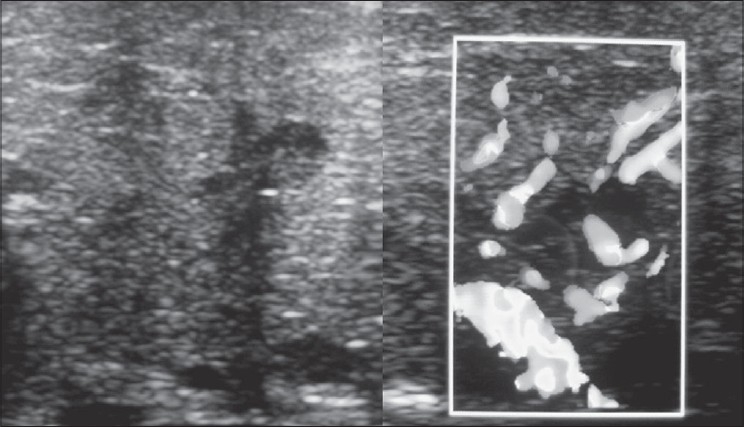
Ultrasound of the parotid gland showing diffuse swollen gland with hypoechoic areas (left) and increased vascularity (right)

A nine-year-old male child presented with history of fall of bicycle on him while he was playing. He had transient loss of consciousness and had multiple episodes of vomiting. There was no history of seizures or ear, nasal or oral bleed. His general and systemic examination was unremarkable. Neurologically coma scale was E3V5M6 and pupils were bilaterally equal and reacting to light. Facial nerve functions were normal. There were no focal neurological deficits. Local examination revealed swelling over the left parotid region with bruising over it associated tenderness [Figure 1]. There was mild trismus. Skin was intact. Oral cavity examination was normal. Computed tomography showed marked soft tissue swelling involving the left parotid gland [Figure 2]. There was no evidence of any intracranial hematoma or underlying bony injury. Ultrasound with color Doppler diffusely swollen gland with hypoechoic areas and increased vascularity [Figure 3]. With these findings the child was managed conservatively and is doing well at follow-up.

## DISCUSSION

Trauma to the parotid gland parenchyma and duct produces a clinical picture of facial swelling with minimal pain. Diagnosis can be confirmed by computed tomography or sialography.[1] As in the present case computed tomography will show a swollen gland with an adjacent lucent area.[1] Sialograpy was not performed in our case as there was no evidence of salivary leak and also patient was responding to conservative management. Radiological investigations will also rule out the injury to the skeletal structures and facial nerve.[1] As in our case, once the injuries to the facial nerve, skeletal system and ductal structures can be excluded, a conservative approach to management to these patients is recommended.[1,4] Antibiotics are recommended only when there is clinical evidence of infection.[4] When there is no clinical evidence of salivary leak or any associated injuries patients with blunt parotid trauma do not require invasive diagnostic procedures and can be managed conservatively with good outcome.
